# Elevated risk of thrombotic manifestations of SARS-CoV-2 infection in cancer patients: A literature review

**DOI:** 10.17179/excli2022-5073

**Published:** 2022-06-30

**Authors:** Annunziata Stefanile, Maria Cellerino, Tatiana Koudriavtseva

**Affiliations:** 1 Department Clinical Pathology and Cancer Biobank, IRCCS Regina Elena National Cancer Institute, Istituti Fisioterapici Ospitalieri (IFO), 00144 Rome, Italy; 2 Department of Clinical Experimental Oncology, IRCCS Regina Elena National Cancer Institute, IFO, Via Elio Chianesi 53, 00144, Rome, Italy; 3 Department of Neuroscience, Rehabilitation, Ophthalmology, Genetics, and Mother-Child Health (DINOGMI), University of Genoa, Genoa, Italy; 4 Medical Direction, IRCCS Regina Elena National Cancer Institute, Istituti Fisioterapici Ospitalieri (IFO), 00144 Rome, Italy

**Keywords:** SARS-CoV-2, COVID-19, cancer, coagulation, thrombotic manifestations

## Abstract

Coronavirus disease 2019 (COVID-19) results in higher risks of hospitalization or death in older patients and those with multiple comorbidities, including malignancies. Patients with cancer have greater risks of COVID-19 onset and worse prognosis. This excess is mainly explained by thrombotic complications. Indeed, an imbalance in the equilibrium between clot formation and bleeding, increased activation of coagulation, and endothelial dysfunction characterize both COVID-19 patients and those with cancer. With this review, we provide a summary of the pathological mechanisms of coagulation and thrombotic manifestations in these patients and discuss the possible therapeutic implications of these phenomena.

## Background

The new pathogen belonging to the Coronaviridae family (SARS-CoV-2) is known as the seventh beta-coronavirus for infections in humans after the Severe Acute Respiratory Syndrome Coronavirus (SARS-CoV1) and the Middle East Respiratory Syndrome Coronavirus (MERS-CoV), responsible of severe epidemics in 2002 and 2012, respectively (Zhu et al., 2020[81]). SARS-CoV-2 disease 2019 (COVID-19) is the largest pandemic disease of humans in the past century. Up to April 2022, more than 500 million confirmed cases of COVID-19 have been reported worldwide (https://www.worldometers.info/coronavirus). 

The clinical manifestations of COVID-19 can be highly variable, ranging from mild upper respiratory tract symptoms in most cases to severe, life-threatening, multi-organ disease. Overall, the risks of hospitalization or death are higher in older patients and those with underlying conditions. Thrombotic complications are a major cause of morbidity and mortality in patients with COVID-1 (Pollard et al., 2020[59]). Patients with pre-existing cardiovascular disease and/or traditional cardiovascular risk factors, including obesity, diabetes mellitus and hypertension are indeed at the highest risk of death from COVID-19 (Pollard et al., 2020[59]; Gao et al., 2021[20]).

Cancer patients have greater risks of COVID-19 onset and worse prognosis (di Cosimo et al., 2021[13]). Possible explanations for this phenomenon include a systemic immunosuppressive state caused by malignancy and anticancer treatments, older ages and major comorbidities including a propensity to thrombosis and greater contact with the healthcare system. The worldwide mortality rate for cancer patients who develop COVID-19 remains high (25.6 %), even when adequate therapy is administered (di Cosimo et al., 2021[13]; Jing et al., 2022[33]). Taken together, these aspects prompted us to focus on the thrombotic risk in COVID-19 patients with cancer. We herein provide a review of the current literature involving the mechanism of thrombotic processes determined by SARS-CoV-2, focusing on the thrombotic manifestations of the infection in cancer patients.

## Pathological Mechanisms of SARS-CoV-2 Infection

According to current evidence, homotrimers of the spike protein (S) present on the viral envelope played a key role in the pathogenesis of SARS-CoV-2. Protein S binds with high affinity the angiotensin-converting enzyme-2 receptor (ACE2) and promotes virus invasion into target cells (Sungnak et al., 2020[66]). Once inside the target cells, the viral genome is freed from the capsid for replication and translation. Cleavage of Protein S mediated by transmembrane protease serine 2 (TMPRSS2) and endosomal cysteine protease B and L is crucial for the entry of SARS-CoV-2 into the host cell (Hoffmann et al., 2020[29]; Sungnak et al., 2020[66]). Although TMPRSS2 is highly represented in lung tissue cells, its expression changes between different populations determining a different susceptibility to SARS-CoV-2 infections (Mollica et al., 2020[51]). 

Binding of the virus to ACE2 alters the functioning of the renin-angiotensin system (RAS) by decreasing the regulation of ACE2 expression and increasing that of angiotensin II (Ang II) (Walls et al., 2020[75]; Lo et al., 2020[46]). In patients with COVID-19, increased concentrations of Ang II, a potent vasoconstrictor agent, result in increased tissue factor (TF) and plasminogen activator inhibitor-1 (PAI), contributing to the hypercoagulable state (Miesbach and Makris, 2020[49]). 

Single cell RNA viral sequencing studies (scRNA-seq) in human, non-human primate and mouse cell lines respectively, have recognized type II pneumocytes, enterocytes and goblet cells secretory of the nasal mucosa as major targets of SARS-CoV-2 (Ziegler et al., 2020[82]). These studies have also shown that the expression of the gene encoding the ACE2 protein is mainly induced by interferon (IFN); specifically, in the nasal epithelium and bronchial cells, the upregulation of ACE2 is mediated by IFN-α. SARS-CoV-2 exploits the overexpression of ACE2 receptors on the surface of the lung epithelium to facilitate viral infection thus increasing the probability of poor prognosis in patients with SARS-CoV-2. The expression of ACE2 mRNA in vascular endothelial cells of the lung, extrapulmonary tissue, heart, nervous system, intestine, kidneys, blood vessels, muscles, makes us understand how COVID-19 is associated with the damage of many organs and tissues (Ziegler et al., 2020[82]). 

It has also been shown that binding of protein S to CD147 transmembrane glycoprotein present on activated T lymphocytes results in severe lymphopenia in COVID-19 patients (Helal et al., 2022[27]). Based on the evidence of this interaction, a recent study suggested that CD147 could be a future target for drug treatment to improve the prognosis of COVID-19 patients (Helal et al., 2022[27]). 

First, SARS-CoV-2 alters the morphology of lung cells compromising lung function, in a second stage progressive hyperinflammation contributes to the development of alterations in the hemostatic system which increases the risk of serious systemic complications such as thromboembolic events, as described in the following paragraphs (Hamming et al., 2004[25]).

## Coagulation in COVID-19 Patients with Cancer

### Pathological mechanisms of coagulation and thrombotic manifestations in COVID-19

It is well known that the rapid viral replication of SARS-CoV-2 in the host induces a massive production of cytokines and chemokines, and that this process is closely related to alterations of the hemostatic system (Henry et al., 2020[28]). Inflammatory status and RAS dysfunction during SARS-CoV-2 infection leads to acute pulmonary vascular damage. Consequently, the resulting hypoxia alters the lung endothelial cells, thus leading to the reduction of anticoagulant molecules and the increase of procoagulant ones, favoring the activation of the immune-thrombosis process (Henry et al., 2020[28]). This process initiates the formation of micro-clots in the blood circulation of COVID-19 patients and contributes to the extent of damage up to lung collapse (Henry et al., 2020[28]).

The intricate and close relationship between the coagulation system and the immune system is the key to understand the immune-thrombotic process. As a result of SARS-CoV-2 induced inflammation, activated platelets exert their pro-inflammatory effects, mainly through the secretion of IL-1β and the overexpression of membrane glycoproteins P-selectin on their surface which are used for the recruitment of polymorphonuclear leukocytes (PMN) (Morrell et al., 2014[53]). The production of platelet-neutrophil complexes can generate vaso-occlusive thrombi and multiorgan damage. To enhance the processes of immune-thrombosis are components of the classical complement pathway as C3a and C5a fragments with a series of events such as the further mobility of PMNs, mast cell degranulation and increased exposure of TF expression on endothelial and platelet cells and von Willebrand factor (VWF) multimers respectively (Keragala et al., 2018[35]). Experiments conducted in SARS models demonstrated mild identity lung lesions in C3 knockout mice (C3 -/-) almost comparable to control mice. This means that disorders of the innate immunity contribute to the immunopathology that characterizes SARS-CoV-2 induced disease and that inhibition of the activation of systemic inflammation-reducing complement could suggest a possible immunotherapy treatment in patients with SARS-CoV-2 (Gralinski et al., 2018[23]). 

The multimers of VWF circulating in the blood, after secretion and anchorage to endothelial cells, bind to platelets, resulting in microthrombi. This condition in patients with COVID-19 has resulted in microangiopathy. Under physiological conditions the VWF are cleaved by a disintegrin and a metalloproteinase secreted in the blood called ADAMTS13 with a thrombospondin motif type 1 member 13, also known as the VWF cleavage protease (VWFCP) which decreases the activity of VWF. The role of ADAMTS13 in this context is controversial, since one study found no evidence of a biological role of the protein (Levi et al., 2020[43]). Unlike this study, recent research showed that the reduction in the concentration of the ADAMTS13 protein causes thrombus and a reduction, albeit highly variable, in the number of platelets, being ADAMTS13 deficiency associated with a greater risk of mortality during hospitalization of patients with COVID-19 (Tiscia et al., 2020[70]). 

The complement proteins involved in the mannose-binding lectin (MBL) pathway, in particular the MBL-associated serine protease 1 and 2 (MASP-1 and MASP-2), also contribute to the hypercoagulability state (Keragala et al., 2018[35]). MASP-1 initiates the MBL pathway by cleavage of prothrombin and its role in thrombogenesis both *in vitro *and* in vivo* is known from scientific evidence. MASP-2 acts with proteolytic activity on C4 and C2 generating the C3 convertase (Jenny et al., 2015[32]). MASP-2 acts with proteolytic activity on C4 and C2 generating the C3 convertase. Platelets in thrombotic disorders activate MASP-1 and MASP-2 by upregulating coagulation and inflammation processes (Kozarcanin et al., 2016[39]). A recent study (Eriksson et al., 2020[16]) showed higher plasma MBL levels in a subgroup of 65 COVID-19 patients with thromboembolism (TE) undergoing intensive care compared to patients who did not have TE. Increased values of MBL correlated with high plasma levels of D-dimer, suggesting that MBL could favor the thrombotic event in severe COVID-19 patients. Therefore, MLB could be evaluated as a new risk marker for TE and considered as a possible candidate for future treatment strategies in SARS-CoV-2 patients (Eriksson et al., 2020[16]). 

Other studies also include the neutrophil extracellular traps (NET) among future therapeutic targets for the treatment of SARS-CoV-2 (Middleton et al., 2020[48]; Gollomp et al., 2018[22]). NET, consisting of neutrophil DNA and histones, are poured into the extracellular environment by neutrophils when T lymphocytes release TNF, IL-17 and IFN gamma. The neutrophils involved in the front line in the defense of our organism, in the pathogenesis of SARS-CoV-2, change their role and become allies of the virus (Jing et al., 2022[33]) . NETs have already been studied in the past for their highly thrombogenic power and represent increasingly important entities to study the immune-thrombotic process. NETs exert their procoagulant power through numerous biological mechanisms. They have a direct action on both factor XII (FXII) and tissue factor (TF) by activating the intrinsic and extrinsic coagulation pathway respectively, they bind to VWF causing platelet capture, release neutrophil elastase, cathepsin G, myeloperoxidase, events that further contribute to the formation of intravascular thrombus (Jing et al., 2022[33]; Middleton et al., 2020[48]; Gollomp et al., 2018[22]). They also cleave physiological anticoagulants such as tissue factor inhibitor TFPI and thrombomodulin and histones H3 and H4 of NETs act as DAMP and activate complement system pathways (Gollomp et al., 2018[22]; Jing et al., 2022[33]; Middleton et al., 2020[48]). Recently, numerous studies have considered NETs as prognostic and survival indicators in COVID-19 patients given the presence of NETs in the serum of severe COVID-19 patients. A prospective study of 33 COVID-19 patients and 17 age- and sex-matched controls revealed that an increase circulating NETs in plasma are related to more severe clinical manifestations of respiratory disease (Middleton et al., 2020[48]). Indeed, elevated levels of NETs were observed in tracheal aspirates from intubated versus non-intubated COVID-19 patients. Autopsy examinations on the lungs of COVID-19 patients revealed microvascular thrombi demonstrated by the co-localization of platelet aggregates with neutrophil histones (Middleton et al., 2020[48]). The binding of the anti-heparin platelet factor 4 (PF4) released by the α granules of the platelets to the NETs contributes to make the latter resistant to DNase and this explains the high levels of PF4 and NET in COVID-19 patients (Gollomp et al., 2018[22]). 

To date, there are no specific laboratory markers capable of evaluating COVID-19 but what emerges from the numerous studies in patients with the new infection is the alteration of coagulation parameters (Han et al., 2011[26]; Tang et al., 2020[67]). In patients with more severe manifestations of SARS-CoV-2, extremely high D-dimer values reflect coagulopathies and are associated with poor prognosis. A study of 183 Chinese patients during their hospital stay with COVID-19 infection analyzed the differences in coagulation tests between survivors and non-survivors every 3 days for 14 days. Study results showed a significant increase in D-dimer concentrations and fibrin degradation products (FDP) in non-survivors as compared to survivors, with 71.4 % and 0.6 % of the non-surviving and surviving patients meeting the criteria of disseminated intravascular coagulation, respectively (Tang et al., 2020[67]). The same study reported that high concentrations of fibrinogen characterized COVID-19 patients (Tang et al., 2020[67]). Although quantification of plasma fibrinogen is not routinely performed in some laboratories, it can be useful for monitoring the state of hypercoagulability especially in cases where instrumental tests are not easily accessible. Research has highlighted the clinical importance of thrombocytosis in COVID-19 patients. The administration of antiplatelet drugs and low molecular weight heparin have underlined the decidedly beneficial effects in the most severe phenotypes of SARS-CoV-2 infection (Ranucci et al., 2020[61]). 

Other studies have shown that thrombocytopenia is associated with more severe clinical manifestations of SARS-CoV-2 and a high mortality rate (Lippi et al., 2020[45]). Lippi et al. (2020[45]) conducted a meta-analysis of studies reporting relative data on number of platelets in 1779 patients with SARS-Co-V2. Based on the calculation of the weighted mean difference (WMD) of platelets, they identified serious patients with COVID-19 from non-serious ones and revealed that the most severe manifestations are associated with thrombocytopenia. These results point at platelets as useful and quick marker to predict a poor prognosis in SARS-CoV-2 infected patients during hospitalization (Lippi et al., 2020[45]). In conditions of severe pulmonary insufficiency, several mechanisms may contribute to platelets reduction. Lung endothelial damage caused by viral infection and mechanical ventilation triggers platelet activation and aggregation, resulting in pulmonary thrombus formation and extensive platelet consumption (Yang et al., 2005[78]). Inflammation, long-term ventilation, and/or oxygen therapy can cause changes in the pulmonary capillary bed. Since the lung can be a release site for platelets derived from the fragmentation of precursors called megakaryocytes, changes in the capillary bed can lead to an imbalance in platelet defragmentation. Furthermore, coronaviruses can directly affect blood cells and trigger an autoimmune response or alter the normal hematopoietic process in the bone marrow (Yang et al., 2005[78]). 

Several studies have shown the development of serious thrombotic complications in COVID-19 patients. A high incidence of deep venous thromboembolism (VTE) in patients who died with COVID-19 emerged from a prospective autopsy study, where in 33.3 % of cases pulmonary embolism (PE) was identified as the defining event of death (Wichmann et al., 2020[76]). In a retrospective study of 71 COVID-19 patients hospitalized and undergoing thromboprophylaxis, it was found that 22.5 % and 10 % had VTE and PE, respectively, being D-dimer concentrations positive predictors of thrombotic events (Artifoni et al., 2020[4]). Cui et al. (2020[11]) reported a 25 % incidence of VTE in 81 patients with severe SARS-CoV-2 pneumonia in the intensive care unit (ICU), 40 % of which had a fatal outcome, with D-dimer concentrations being predictive of clinical outcome. Furthermore, according to the authors, D-dimer could be considered a marker to test the efficacy of anticoagulant therapy associated with a better clinical prognosis (Cui et al., 2020[11]). In this regard, Escher et al. (2020[17]), reported a reduction in the concentration of D-dimer in a patient who underwent an enhanced anticoagulant dose (Escher et al., 2020[17]). Another study by Klok et al. (2020[36]) explored the incidence of coagulopathies in a cohort of 184 hospitalized and ICU patients with SARS-CoV-2 pneumonia. PE was the most frequently encountered coagulopathy in these patients defined by prolongation of prothrombin time (PT)>3 seconds and prolongation of activated partial thromboplastin time (aPTT)> 5 seconds. These coagulation parameters independently predicted thrombotic complications (Klok et al., 2020[36]).

### Pathological mechanisms of coagulation and thrombotic manifestations in cancer patients

Cancer cells can alter the hemostasis of plasmatic coagulation, circulating platelets and vascular endothelium, thereby causing an imbalance in the equilibrium between clot formation and bleeding (Bauer et al., 2022[5]). Thrombotic complications not only correlate with disease progression and prognosis, but may also be suggestive of an occult cancer (Monreal et al., 2004[52]). Indeed, tumor cells' surfaces are characterized by the expression of procoagulant factors, such as TF (Bauer et al., 2022[5]). A high TF expression in tumor tissue of cancer patients has been correlated with tumor progression, worse prognosis, and thrombosis (Rondon et al., 2019[63]). In response to blood exposure, TF interacts with plasmatic coagulation factors and promotes the generation of thrombin, which can exert its thrombotic effects via cleavage of fibrinogen and exhibits pleiotropic cellular effects mediated through protease activated receptors (PARs) (Bauer et al., 2022[5]; Han et al., 2011[26]). Thrombin has been shown to activate human platelets via PAR-1 and PAR-4, resulting in the secretion of a wide range of molecules (including matrix metalloproteinases, platelet factor 4 and VEGF) (Han et al., 2011[26]). Therefore, tumors may use the proangiogenic properties of platelets for the formation of new blood vessels, supporting the migration and the activation of endothelial cells (Ruf, 2007[64]). Cancer cells overexpressing PARs result in enhanced metastasis in mouse model (Bauer et al., 2022[5]). Thus, TF on the cell surface could represent a mechanistic interconnection between hemostasis and tumor progression (Bauer et al., 2022[5]). 

In parallel, it has been demonstrated that tumor cell-generated thrombin or tumor cell-secreted VEGF-A can induce endothelial cell transformation into an adhesive and procoagulant surface, through the release of molecules such as interleukin-8, Ang-2, P-selectin and VWF (Bauer et al., 2022[5]; Wagner and Frenette, 2008[74]). In this context, selectins are considered the initial mediators for the recruitment of leukocytes to the vessel wall (Läubli and Borsig, 2010[42]), while experimental studies revealed that VWF networks act as linker for adhesion of monocytes and neutrophils to the vascular endothelium supporting extravasation of leukocytes and therefore inflammation (Läubli and Borsig, 2010[42]; Pendu et al., 2006[56]; Bauer et al., 2022[5]). Of those, neutrophils seem to play a critical role for malignancy, coagulation, and inflammation (Bauer et al., 2022[5]; Koudriavtseva et al., 2021[38]). Interestingly, it has also been shown that NETs - whose thrombogenic role has already been described in detail in the context of COVID-19 - are able to directly trap circulating tumor cells and support extravasation and thus metastasis (Wu et al., 2019[77]). Clinical trials provide evidence that elevated levels of NETs can be used as biomarkers to detect early tumors and predict the development of metastases (Decker et al., 2019[12]). Moreover, NETs are well-known risk factors for tumor-associated vascular dysfunction, thrombosis, and organ-failure (Bauer et al., 2022[5]). Thus, the close interaction between the innate immune system and the coagulation suggests overlapping mechanisms of hemostasis and inflammation in cancer (Engelmann and Massberg, 2013[15]).

Similarly to what already described in COVID-19 patients, the complement system has also gained increased attention as major contributor to immune-thrombosis under pathologic conditions as manifested in malignancy (Bauer et al., 2022[5]). Complement activation has been reported in ovarian (Bjørge et al., 2005[6]) cancer and brain tumors (Bouwens et al., 2015[7]), and was proposed as a prognostic marker for patients with lung (Ajona et al., 2013[3]) and colorectal cancer (Ytting et al., 2008[79]). Of note, upregulation of the complement C3a receptor was shown to activate neutrophils followed by induction of NETosis and thrombosis and to promote tumor progression (Guglietta et al., 2016[24]).

Despite considerable progress in the treatment of cancer using targeted therapies and immune checkpoint inhibition (ICI), management of cancer patients remains challenging (Bauer et al., 2022[5]). Thrombotic events remain a major complication in malignancy. The incidence of VTE and PE in patients with cancer without SARS-CoV-2 infection is increased of 5 to 7 times and 3 times, respectively, compared to the normal population (Horowitz and Brenner, 2020[30]). Recent evidence confirms that the risk for the development of cancer-associated thrombosis is increasing steadily and is 9-fold higher than in the general population (Mulder et al., 2021[54]). Adelborg et al. (2019[2]) reported data regarding a Danish cohort of over 32000 patients, and showed that approximately 2 of 10 hematological cancer patients experienced myocardial infarction, ischemic stroke, VTE, or bleeding requiring hospital contact within 10 years after hematological cancer diagnosis (Adelborg et al., 2019[2]). Similarly, Navi et al. (2017[55]) evaluated the onset of arterial TE events in patients with malignant tumors and demonstrated a rate of TE twice as high as controls (4.7 % vs 2.2 %) (Navi et al., 2017[55]). Several previous studies suggested that the prevalence of thrombotic events in cancer patients depends mainly on the tumor entity, the disease stage, the tumor grade, location of metastases, the body mass index, and the anti-tumor therapy (Bauer et al., 2022[5]). It has also been demonstrated that the risk to develop VTE is higher in cancer patients receiving chemotherapy or targeted therapy. Further, VTE has been recently suggested as an underappreciated and important immune-related adverse event associated with cancer immunotherapy. Cancer patients under ICI therapy are indeed at high risk for the development of cancer associated thrombosis with increased mortality rates (Icht et al., 2021[31]).

### Risk of thrombosis in COVID-19 patients with cancer

**Figure 1:**
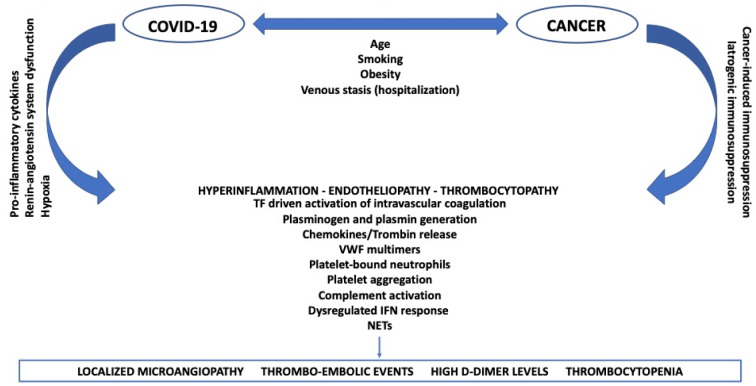
Mechanisms of thrombosis in SARS-CoV-2 infected patients with cancer: a synergic process COVID-19: coronavirus disease 2019; TF: tissue factor; VWF: von Willebrand factor; IFN: interferon; NETs: neutrophil extracellular traps

The mechanisms of thrombosis in SARS-CoV-2 infected patients with malignancy are not separated, but a result of the synergy between COVID-19 and cancer affecting the same coagulation system, as described above and shown in Figure 1(Fig. 1). Alterations in blood flow, increased activation of coagulation and endothelial dysfunction - together with venous stasis which characterize hospitalized patients - are considered the main risk factors for both cancer patients and those with COVID-19.

The inflammatory state triggered by SARS-CoV-2 infection and enhanced by cancer (Falanga et al., 2015[18]) contributes to the development of coagulopathies. Systemic inflammation in SARS-CoV-2 patients is caused by a storm of cytokines such as tumor necrosis factor-α (TNF-α) and interleukins (IL) such as IL-6, IL-1, IL- 1b -17a which induce the expression of tissue factor on mononuclear cells and VWF on vascular endothelial cells (Jose and Manuel, 2020[34]), leading to elevated concentrations of the fibrinolysis inhibitor plasminogen activator-1 and contributing to both blood clot and platelet hyperactivation and a dysregulation of the normal anticoagulant pattern, through the downregulation of thrombomodulin expression (Levi et al., 2020[43]; Dosquet et al., 1995[14]). In addition, an increase in IL-2, IL-7, granulocyte colony-stimulating factor (G-CSF), interferon-inducible protein 10 (IP-10), monocyte chemotactic protein 1 (MCP-1), macrophage inflammatory Protein-1α (MIP-1α) characterize the more severe phenotypes of COVID-19 (Mehta et al., 2020[47]). Phospholipids produced by oxidative stress make a significant contribution to viral inflammation and have been found in the lungs of patients with severe SARS-CoV-2. TF and NETs are also very important components in promoting the activation of coagulation and angiogenesis in both solid and hematological tumors (Falanga et al., 2015[18]; Thålin et al., 2019[69]), but may also be related to thrombosis in severe COVID-19, as previously described. Additionally, cancer patients exhibit impaired innate immunity and dysregulated IFN response (Falanga et al., 2015[18]). In parallel, SARS-CoV-2 has evolved diverse mechanisms to evade host antiviral responses, which can prevent the signaling pathway of endogenous IFN induction. Therefore, COVID-19 patients with cancer manifest lower levels of IFN-I and IFN-III, moderate IFN stimulated genes, and proinflammatory cytokine production, which indicates decreased antiviral immune function (Perico et al., 2021[57]). Interestingly, evidence suggests that virus inhibited IFN synthesis and high levels of proinflammatory cytokines are correlated with increased disease severity (Brain et al., 2021[8]).

Since the beginning of the pandemic, Liang et al. (2020[44]) stated that cancer patients with COVID-19 have a 3.5-time greater risk of undergoing artificial respiration, intensive care, or death than patients without cancer. In patients with solid and blood cancers, reduction of both neutrophils and lymphocytes, damage to natural defense barriers such as the nasal mucosa, skin, altered splenic and humoral function, bone marrow disorders and anticancer treatments worsened the outcome of SARS-CoV-2 infection (Liang et al., 2020[44]; Rogado et al., 2020[62]). Anti-cancer drug treatments such as ICI, bispecific T cell engagers (BiTE), cytokines, chimeric T receptor cells (CAR-T) and allogeneic stem cell transplantation, can cause harmful immune-mediated reactions increasing the inflammation within of the pulmonary alveoli (Postow et al., 2018[60]). It has been shown in fact that SARS-CoV-2, unlike other acute respiratory syndromes, could contribute more rapidly to the structural and functional worsening of the pulmonary vascular endothelium (Ackermann et al., 2020[1]). The endothelial damage of the blood vessels of the pulmonary alveoli affected by SARS-CoV-2 has been evidenced by the intracellular localization of the virus and by the rupture of the membranes of the endothelial cells. Diffuse thrombosis with microangiopathy and vessel occlusion characterized the pulmonary vascular system in patients who died from COVID-19 (Ackermann et al., 2020[1]).

Several studies suggested that risk factors such as older age, smoking habits, pre-existing comorbidities, cancer treatments are the main variables associated with the severity of the clinical manifestations of COVID-19 and the worsening of the prognosis of the infection in cancer patients. The first Chinese epidemiological study of 31 January 2020 on the incidence of cancer patients with COVID-19 infection was conducted by Liang and his collaborators (Liang et al., 2020[44]). They evaluated a cohort of 1,590 COVID-19 cases and revealed that 1 % of hospitalized patients had cancer. This percentage was higher than the incidence of cancer in the general Chinese population (0.29 %). Another study conducted by Horowitz and Brenner (2020[30]), performed on a cohort of 1,524 patients admitted to the Wuhan hospital, showed an incidence of COVID-19 infections of 0.79 % and 0.39 % respectively in patients with and without cancer. Both these studies show an association between higher risk of infection and increased age. Similarly, Garassino et al. (2020[21]) showed that an older population with a history of smoking and thoracic cancer is more susceptible to SARS-CoV-2 infection. They analyzed data provided by the TERAVOLT registry (Thoracic cancer international COVID-19 collaboration), including 200 active or former smokers with thoracic neoplasm undergoing cancer treatments. Authors report that patients with stage IV NSCLC lung cancer and aged 61-75 years had more severe COVID-19 phenotypes. Interestingly, 33 % of mortality appeared to be attributable to the history of smoking alone, pointing at smoke as a risk factor for the severity of COVID-19 in cancer patients. Similar results were obtained by Kuderer et al. (2020[40]), who evaluated the association between prognostic variables with mortality within one month of SARS-CoV-2 infection in cohort of 928 cancer patients of different ethnicities and races. They observed that age, male sex, comorbidities, and smoking were the main causes of death, and that active cancers were related to severe COVID-19. It is well documented that smoking is one of the factors most directly implicated in the pathogenesis of inflammatory respiratory diseases such as chronic obstructive pulmonary disease (COPD), bronchitis, asthma, and is the cause of high mortality for emphysema and lung cancerCDC, 2010[9]). An increase in the expression of the ACE2 on the alveolar cells of the lungs of smokers and a consequent increase in mucus secretion by goblet cells has been shown in human and animal models; this can further explain the worse clinical condition of the cancer patients affected by COVID-19 with more aggressive acute respiratory syndrome (Vaduganathan et al., 2020[72]). Subsequent reports confirmed the high vulnerability of cancer patients when infected with SARS-CoV-2. Recently, an ambispective study encompassing 17 hospitals in France, showed that patients with cancer and requiring ICU admission for COVID-19 had an increased mortality, hematological malignancy harboring the higher risk in comparison to solid tumors (Plais et al., 2022[58]). Many risk factors are known to influence infection severity in cancer patients, including delayed admission, low sensitivity, or wrong interpretation of the SARS‐CoV‐2 RT‐PCR tests, cancer treatment (including chemotherapy, targeted therapy, radiotherapy, immunotherapy, and treatment regimens containing JAKi or BTKi, as well as treatment with high‐dose corticosteroids and ICIs). In some cases, the initial diagnosis may not be correct due to the similarity of cancer symptoms and COVID‐19 (Mohseni Afshar et al., 2022[50]). 

## Therapeutic Implications

In cancer patients with SARS-CoV-2 infection, the anticancer treatment has raised critical issues (Ueda et al., 2020[71]). In the United States, the decision to discontinue anticancer treatment in cancer patients during the COVID-19 pandemic has been a difficult decision since early cases of SARS-CoV-2 infection due to the benefit/risk assessment that such discontinuation entailed (Mohseni Afshar et al., 2022[50]). Delays in anticancer treatments, particularly at the first diagnosis of cancer or the onset of metastases, could heavily affect the prognosis of the disease (Ueda et al., 2020[71]). On the other hand, the use of anticancer therapy during 14-day period before SARS-CoV-2 infection diagnosis has been shown to be a negative prognostic factor for its outcome in cancer patients (Zhang et al., 2020[80]). Patients who underwent chemotherapy or surgery 30 days before the onset of COVID-19 have been also more susceptible to this infection than patients without anticancer treatments and surgery (Liang et al., 2020[44]). The specifications provided by Kutikov et al. (2020[41]), recommend delaying chemotherapy treatment during the pandemic in patients at low cancer risk. Overall, it is recommended that cancer patients be thoroughly screened for COVID-19 infection to avoid immunosuppressive treatment or to reduce its dosage in case of a positive COVID-19 finding (Kutikov et al., 2020[41]).

In addition to anti-cancer treatments, alternative therapeutic approaches to ameliorate symptoms and delay disease progression should be used. In the context of a prethrombotic state of cancer, together with extensive endothelial cells damage and activation, and along with COVID-19 induced hyperinflammation contributing to the development of alterations in the hemostatic system, effective anticoagulant therapeutics are highly warranted (Jing et al., 2022[33]; Salabei et al., 2021[65]). Several studies suggest that thrombotic risk should not be underestimated to ensure better management of SARS-CoV-2 infection. Administration of the low molecular weight heparin anticoagulant (LMWH) appears to contribute to clinical improvement in patients with high D-dimer values and with more severe forms of COVID-19 (Tang et al., 2020[67]). Drug treatment with heparin in the presence of coagulopathy associated with elevated D-dimer levels was effective and decreased mortality in SARS-CoV-2 patients by 20 % (Thachil, 2020[68]). The anti-inflammatory properties of heparin have been demonstrated in experimental models of immune-thrombosis through inhibition of thrombin function, binding to inflammatory cytokines, blocking the recruitment of neutrophils and leukocytes and neutralization of the complement peptide factor C5a (Gaertner and Massberg, 2016[19]). The necessary condition for the new coronavirus to attack the ACE2 receptor in the lungs is the binding of the virus with the heparan sulfate carbohydrate present on the cell surface and on the pulmonary capillary endothelium (Clausen et al., 2020[10]). In this regard, heparin was discovered to be a new therapeutic approach for COVID-19 infection, as the specific binding of the anticoagulant to the carbohydrate blocks the attack of SARS-CoV-2 on ACE2 receptor, thus preventing viral entry into the cell (Clausen et al., 2020[10]). Although direct-acting oral anticoagulants (DOACs) are an effective treatment option for patients with cancer and acute VTE because they inhibit cancer-induced prothrombotic state, current clinical guidelines recommend LMWH as the first-line treatment of short- and long-term cancer-related VTE (Jing et al., 2022[33]), and data regarding DOACs use in COVID-19 patients with COVID-19 are still lacking. Data on antithrombotic treatment regimens in COVID-19 patients mostly derive from retrospective studies, many with a small sample size (Jing et al., 2022[33]; Salabei et al., 2021[65]). Therefore, current recommendations on screening, diagnosis, and treatment of COVID‐19-associated thrombosis and coagulopathy are mostly based on expert opinion consensus. Overall, the prevention of thromboembolic complications is recommended for severe COVID-19 illness even after discharge (Jing et al., 2022[33]). However, a risk assessment depending on patients' clinical characteristics (i.e. age, smoke use, comorbidities, cardiovascular risk factors) and the severity of the disease is warranted for each patient. Another important factor seems to be the optimal timing of thromboprophylaxis, with the best beneficial effects expected when administered before the development of critical illness (Kollias et al., 2021[37]). In accordance with the evidence that COVID-19 and cancer are both conditions leading to an hyperinflammatory state that makes patients more prone to thrombotic events, it is plausible that anti-inflammatory and anticoagulant therapy may be more effective than either medicine alone in the prevention of thrombosis and death. In this context, several treatments have been proposed to reduce the risks of COVID-19 associated complications or death, including the IL-6 receptor antagonist tocilizumab or the IL-1β receptor antagonist anakinra (Jing et al., 2022[33]; van de Veerdonk et al., 2022[73]). Given the dynamic of Sars-CoV-2 infection - in order to allow the formation of effective immunity in the first phases of COVID-19 - the optimal timing for anti-inflammatory therapy may be at the first signs of respiratory distress (van de Veerdonk et al., 2022[73]; Jing et al., 2022[33]). As per corticosteroids' administration, it is worth noting that - since cancer-mediated immune suppression coupled with dexamethasone inhibits the production of effective antibodies, delaying viral clearance and aggravating disease progression - dexamethasone is not recommended in COVID-19 patients with cancer (Jing et al., 2022[33]).

## Conclusions

In conclusions, thrombotic complications are considered a major cause of morbidity and mortality in patients with COVID-19. Cancer is a severe condition known to increase risk of death from COVID-19, as thrombotic events are commonly observed in patients with malignancies. Accordingly, a deep knowledge of mechanisms leading to these phenomena together with a prompt and adequate management of COVID-19 patients with cancer appears of paramount importance in order to improve prognosis of these patients. This review, by examining an extensive literature, has summarized the pathological mechanisms of coagulation and thrombotic manifestations in COVID-19 patients and those with cancer. We highlighted the role of systemic inflammation and consequent immune-thrombosis in this context, showing that these phenomena are not separated, but a result of the synergy between COVID-19 and cancer affecting the same coagulation system. We have also discussed the possible therapeutic strategies which should be carried out to reduce the mortality and the risk of sequelae. Considering the increased risk of thrombosis in cancer patients and the potential for coagulation disturbances in the pathophysiological mechanisms of many diseases, we suggest that further studies addressing the pathological mechanism behind these processes and their interactions, exploring the reasons of heterogeneity between patients, and evaluating the applications and timing of different treatment strategies in patients even in the outpatient setting are highly warranted.

## Declaration

### Conflict of interest

The authors declare no conflict of interest.

### Authors' role and contribution

Annunziata Stefanile: major role in the revision of the literature; drafted the manuscript. Maria Cellerino: revision of the literature; drafted the manuscript. Tatiana Koudriavtseva: designed and conceptualized the study; revised the manuscript for intellectual content.
